# Supplementary material for: Inter-hemispheric synchroneity of Holocene precipitation anomalies controlled by Earth’s latitudinal insolation gradients
Source: Nat Commun. 2020 Oct 28;11:5447. doi: 10.1038/s41467-020-19021-3 (PMC7595035; doi:10.1038/s41467-020-19021-3)
Supplement: Supplementary file 1 — Supplementary Information [file 41467_2020_19021_MOESM1_ESM.pdf]

# Supplementary Information

to

# Inter-hemispheric synchroneity of Holocene precipitation anomalies controlled by Earth's latitudinal insolation gradients

*Deininger et al.*

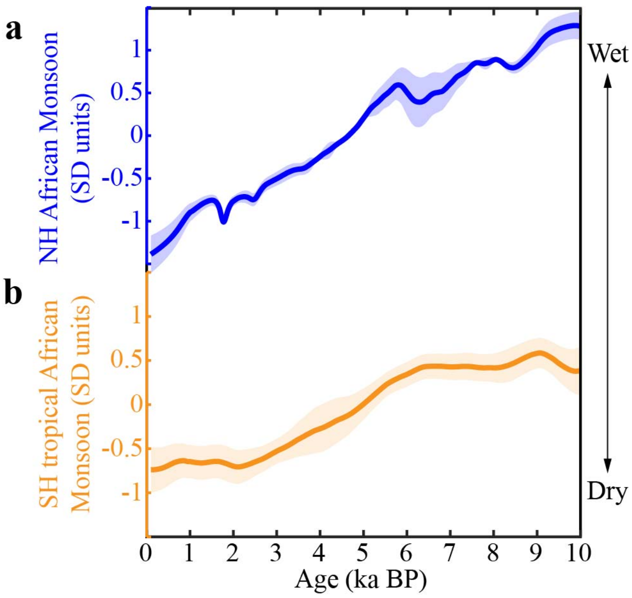


**Supplementary Figure 1. Standardised precipitation changes analysed for two subsets of African hydroclimate records. a,** African Monsoon composite hydroclimate record (stated in standardised (SD) units) calculated from individual hydroclimate records located in the Northern Hemisphere (NH) (>0°latitude) and **b,** from tropical regions on the Southern Hemisphere (SH). The locations of the individual records for these two subsets are illustrated in Figure 2.

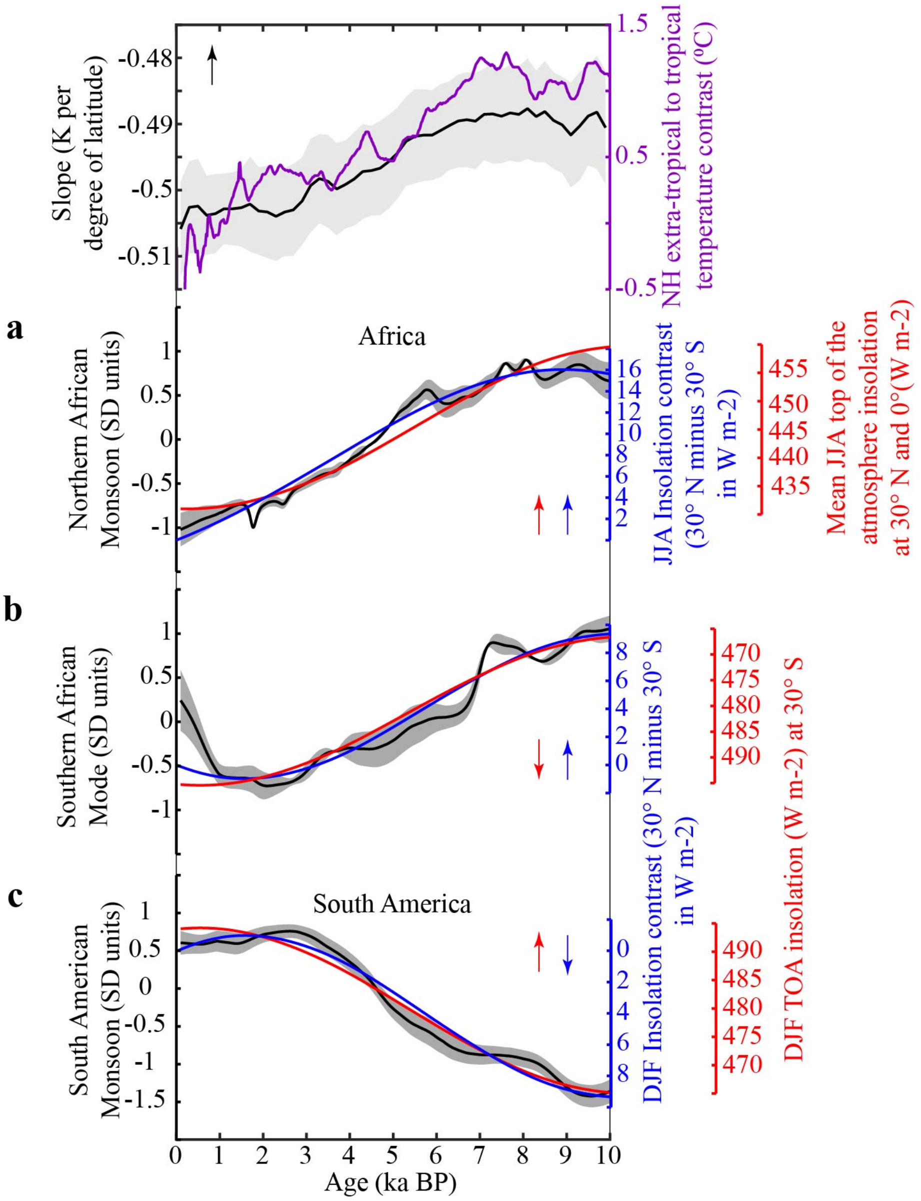


**Supplementary Figure 2. Comparison between hydroclimate changes and the inter-hemispheric insolation contrast as well as with local insolation. a,** reconstructed slope of the Northern Hemisphere (NH) temperature gradients between the low- and high-latitudes (black, ref 1) and the temperature contrast (purple, ref. 2) calculated from zonal averages for the tropical region (30°S to 30° N) and the Northern Hemisphere (NH) extra-tropics (>30 °N). The shading indicates the 1-sigma standard deviation of the slope. **b,** comparison of hydroclimate changes of the African Monsoon (black, the grey shading indicates the 1-sigma standard deviation) with the mean inter-hemispheric insolation contrast (blue) between 30 °N and 30 °S and the mean top-of-the-atmosphere (TOA) insolation (red) at 30°N and 0°during boreal summer (June to August, JJA). The mean top-of-the-atmosphere (TOA) insolation at 30 °N and 0° is used, because of the latitudinal distribution of the used hydroclimate records (Figure 2). Changes in the insolation contrast are given relative to present day. **c,** same as panel b, but for the southern African precipitation dipole and for mean austral summer insolation quantities (December to February, DJF). **d,** same as panel b, but for the hydroclimate changes of the South African Monsoon and for mean austral summer insolation quantities (December to February, DJF). The arrows point in the direction of increasing insolation quantities and an increasing slope (i.e. a weaker) latitudinal temperature gradient.

**Supplementary References**

1 Routson, C. C. et al. Mid-latitude net precipitation decreased with Arctic warming during the Holocene. Nature 568, 83-87 (2019).

2 Marcott, S. A., Shakun, J. D., Clark, P. U. & Mix, A. C. A reconstruction of regional and global temperature for the past 11,300 years. Science 339, 1198-1201 (2013).
