# Supplementary material for: Inter-hemispheric synchroneity of Holocene precipitation anomalies controlled by Earth’s latitudinal insolation gradients
Source: Nat Commun. 2020 Oct 28;11:5447. doi: 10.1038/s41467-020-19021-3 (PMC7595035; doi:10.1038/s41467-020-19021-3)
Supplement: Supplementary file 3 — Description of Additional Supplementary Files [file 41467_2020_19021_MOESM3_ESM.pdf]

Description of Additional Supplementary Files

File Name: Supplementary Data 1
Description: **Hydroclimate records from Africa used in this study**. Information includes longitude, latitude, location, archive type, proxy, interpretation, link to data repository and the reference. The data file includes also the inferred hydroclimate changes used to calculate the composite records

File Name: Supplementary Data 2
Description: **Hydroclimate records from South America used in this study.** Information includes longitude, latitude, location, archive type, proxy, interpretation, link to data repository and the reference. The data file includes also the inferred hydroclimate changes used to calculate the composite records.
